# Variations in the Use of Outpatient Surgery

**DOI:** 10.1001/jamanetworkopen.2025.24165

**Published:** 2025-07-31

**Authors:** Chi Zhang, Kristine Hanson, Lindsey Sangaralingham, Holly K. Van Houten, Zhi Fong, Yu-Hui Chang, Michael Kendrick, David Etzioni, Elizabeth Habermann, Cornelius Thiels

**Affiliations:** 1 Department of Surgery, Mayo Clinic Arizona, Phoenix; 2 Robert D. and Patricia E. Kern Center for the Science of Health Care Delivery, Mayo Clinic Rochester, Rochester, Minnesota; 3 OptumLabs, Eden Prairie, Minnesota; 4 Department of Quantitative Health Sciences, Mayo Clinic Arizona, Scottsdale; 5 Division of Hepatobiliary and Pancreas Surgery, Department of Surgery, Mayo Clinic Rochester, Rochester, Minnesota

## Abstract

**Importance:**

Identification of factors associated with variation in outpatient surgery may further quality improvement efforts to safely reduce postoperative hospital length of stay nationally.

**Objectives:**

To explore variation in the use of outpatient surgery, incorporating patient, geographic, and hospital factors.

**Design, Setting, and Participants:**

This retrospective cross-sectional study used deidentified administrative claims data from OptumLabs Data Warehouse. Participants included adults who underwent 1 of 10 general, urological, or gynecological operations between January 1, 2015, and June 30, 2021, in the US. Patients who underwent combined procedures or reoperations or had at least 15 Elixhauser comorbidities were excluded. Data were analyzed from July 26 to December 16, 2023.

**Exposure:**

Inpatient or outpatient surgical procedures.

**Main Outcomes and Measures:**

Multilevel logistic regression assessed variation in the use of outpatient surgery rates by hospital characteristics (bed size, presence of trainees, and rural referral center status) and hospital census division, adjusting for patient factors (age, sex, number of Elixhauser comorbidities, year, and rural-urban commuting area). This multilevel model allowed for the sources of variability to be quantitatively attributed to patient characteristics, geography, and hospital characteristics.

**Results:**

A total of 330 424 (72.3%) of 456 954 included patients underwent outpatient surgery. The median age was 54 (IQR, 41-67) years, and of those with data available, most patients were female (268 692 of 414 193 [64.9%]). The likelihood of outpatient surgery varied significantly by hospital census division for all 10 operations (eg, MIS salpingo-oophorectomy range, 29.6%-58.8%; *P* < .001). Variation in hospital census division contributed most to outpatient surgery for 8 of 10 operations compared with other patient and hospital characteristics. Hospital census division contributed the greatest degree to the variation in outpatient simple mastectomy (20.6%) and the least to outpatient open ventral hernia repair (0.7%). Multivariable analysis showed that the odds of outpatient surgery for patients from metropolitan areas were higher for minimally invasive salpingo-oophorectomy (odds ratio [OR], 1.62; 95% CI, 1.34-1.95) and open ventral hernia repair (OR, 1.16; 95% CI, 1.09-1.24). Hospitals with 400 or more beds were independently associated with decreased odds of outpatient surgery compared with hospitals with 50 to 199 beds for 4 of 7 operations (MIS paraesophageal hernia repair [OR, 0.58; 95% CI, 0.47-0.71; *P* < .001]; MIS cholecystectomy [OR, 0.73; 95% CI, 0.68-0.78; *P* < .001]; open ventral hernia [OR, 0.51; 95% CI, 0.46-0.57; *P* < .001]; MIS ventral hernia repair [OR, 0.66; 95% CI, 0.56-0.77; *P* < .001]). The presence of a residency training program was independently associated with increased odds of outpatient surgery for simple mastectomy (OR, 1.35; 95% CI, 1.16-1.58; *P* < .001) and mastectomy with reconstruction (OR, 1.50; 95% CI, 1.27-1.77; *P* < .001) and decreased odds of outpatient surgery for minimally invasive cholecystectomy (OR, 0.96; 95% CI, 0.92-1.00; *P* = .04), open ventral hernia repair (OR, 0.93; 95% CI, 0.86-1.00; *P* = .04), and total thyroidectomy (OR, 0.84; 95% CI, 0.71-1.00; *P* = .04).

**Conclusions and Relevance:**

In this cross-sectional study, significant variation existed in the use of outpatient surgery in the US and appeared to be driven primarily by hospital geography. Addressing these variations may improve the use of resources.

## Key Points

**Question:**

What are the factors associated with the use of outpatient surgery?

**Findings:**

In this cross-sectional study of 456 954 participants, variations in the use of outpatient surgery were associated primarily with geographic location. Lower frequency of outpatient surgery at hospitals in the Northeast and Pacific Coastal regions was not explained by patient- or hospital-related factors.

**Meaning:**

Recognition of geographical variations and providing benchmarking national data may improve use of resources for patients undergoing surgery.

## Teaser

This cross-sectional study describes national trends in outpatient surgery and identifies the variations in outpatient surgery practice based on hospital and patient characteristics in a sample of US adults.

## Introduction

Ongoing discourse about health care in the US has emphasized the need for value-based care.^1,2^ Lower or fixed reimbursement rates coupled with advances in surgical and anesthetic techniques over time have facilitated significant changes in surgical practice, especially in the transition from inpatient care for postoperative monitoring to recovery at home across multiple surgical disciplines.^3^ The surge in outpatient surgery transitions has been substantiated by encouraging data showing improved patient satisfaction, lower nosocomial infection rates, and feasibility.^4,5,6,7,8^ Despite these data, an overnight stay to mitigate and to monitor perioperative risk is at times still recommended. However, standardization of care has been shown to improve patient outcomes and decrease overall cost. For example, use of resources and postoperative complications are decreased in protocolized care after pancreatic surgery.^9^ Standardized sepsis and ventilator bundles have similarly demonstrated improved survival and decreased overall cost of hospitalization.^10,11^ Despite data showing that variations in health care contribute to the overall crescendo of health care cost, there are currently no national benchmark data on the safety of outpatient surgery nor descriptions of the differences that drive variation in use of outpatient surgery. Further, expansion of outpatient surgery across geographic regions and practices also remains poorly characterized.

We herein describe national trends in outpatient surgery and identify the variations in outpatient surgery practice based on hospital and patient characteristics. We hypothesized that outpatient surgery practice variations are driven primarily by surgeon practice preferences based on hospital geographic region. Identification of factors associated with variation in the use of outpatient surgery practice may assist with the development of initiatives aimed at safely reducing postoperative hospital length of stay nationally.

## Methods

### Data Source and Study Population

This retrospective cross-sectional analysis included deidentified administrative claims data from the OptumLabs Data Warehouse (OLDW), which contains longitudinal health information for more than 200 million patients, representing a mixture of ages and geographical regions within the US. The database contains medical and pharmacy claims and enrollment records for individuals with commercial insurance and Medicare Advantage enrollees in a large private US health plan.^12^ Data from the American Hospital Association (AHA) were included to analyze hospital-level variables, as the AHA includes a comprehensive census of US hospitals with detailed characteristics about geographic location, staffing, and infrastructure.^13^ This study was deemed exempt by the Mayo Clinic Institutional Board Review for its use of preexisting deidentified data and followed the Strengthening the Reporting of Observational Studies in Epidemiology (STROBE) reporting guideline.

**Figure 1.**
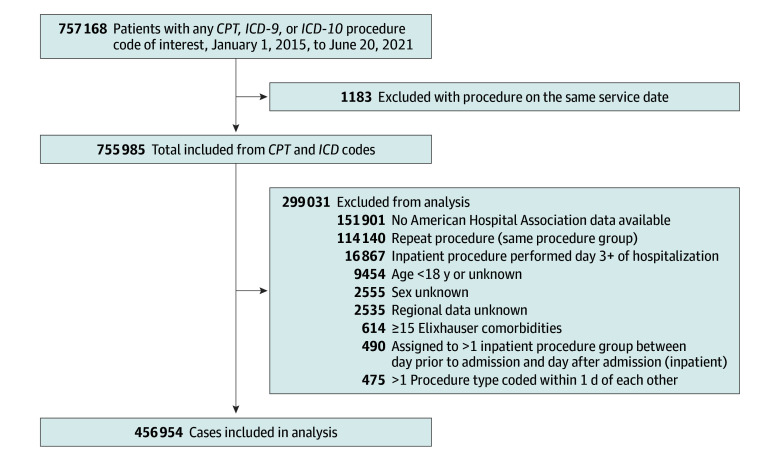
Inclusion and Exclusion Criteria Flow Diagram *CPT* indicates *Current Procedural Terminology*; *ICD-9*, *International Classification of Diseases, Ninth Revision*; and *ICD-10*, *International Statistical Classification of Diseases and Related Health Problems, Tenth Revision*.

We identified adult patients (aged ≥18 years) who underwent 1 of 10 common operations between January 1, 2015, to June 30, 2021. The included operations were simple mastectomy, mastectomy with implant-based reconstruction, minimally invasive (MIS) paraesophageal hernia repair, MIS cholecystectomy, open ventral hernia repair, MIS ventral hernia repair, MIS nephrectomy, MIS hysterectomy, MIS salpingo-oophorectomy (SO), and total thyroidectomy. These operations were chosen because they were the most frequently performed operations within the fields of general surgery, urology, and gynecology in OLDW during the study period.^14,15,16^ Patients were identified in the OLDW database using *Current Procedural Terminology* codes as well as *International Classification of Diseases, Ninth Revision*, and *International and Statistical Classification of Diseases and Related Health Problems, Tenth Revision*, procedural codes (eTable 1 in Supplement 1). Commercial insurance and Medicare Advantage enrollees with medical coverage on the date of the applicable procedure code were included. Patients who underwent concurrent major operations on the same day, more than 1 major operation within 1 day of each other or 1 day prior to admission and 1 day after admission, the same operation within 90 days, or surgery on hospital day 3 or later, who had at least 15 Elixhauser comorbidities, or who had missing demographic information were excluded from the analysis. These criteria were chosen to exclude patients who were unlikely to be realistic outpatient surgery candidates. Patients who underwent surgery at an institution without AHA data were also excluded (Figure 1).

### Covariates and Definitions

Patient factors of interest included year of surgery, age at the time of surgery, sex, Elixhauser comorbidities index, and rural-urban commuting area (RUCA) score. The Elixhauser comorbidities index encompasses 30 comorbidity measures; due to small sample sizes, patients with 8 to 14 Elixhauser comorbidities were combined for analysis.^17^ The RUCA codes classify US census areas by measuring population density and urbanization.^18^ We analyzed RUCA score for the patient’s address at time of surgery as metropolitan vs micropolitan core, small town core, or rural areas, combining the last 3 categories because of small sample sizes.

AHA data on hospital census division and hospital factors, including hospital bed size, presence of a residency training program, and rural referral center identification, were collected.^19,20^ As AHA membership varies by year, if AHA data were not available for the year of surgery, we used available AHA data in the year nearest to surgery to 5 years before or after surgery, with 98% having data within 3 years.

### Outcomes

Outcomes of interest were the rate of outpatient operations for each of the 10 procedures. Patients who had a room-and-board billing charge on the date of surgery or on postoperative day 1 were considered to have undergone inpatient surgery.

### Statistical Analysis

Data were analyzed from July 26 to December 16, 2023. Rates of outpatient surgery were summarized as counts (percentage), and χ^2^ tests were used to compare rates of outpatient surgery across the levels of demographic, clinical, and hospital characteristics. The Cochran-Armitage trend test was performed to evaluate changes in the frequency of outpatient operations over time. Medical coverage was assessed as total number of coverage days in the 365 days prior to the procedure (≤90, 91-182, 183-274, 275-364, and ≥365) and total number of coverage days in the 30 days following the procedure (<30 vs ≥30). Due to the data use agreement with OLDW, counts and percentages were suppressed when sample sizes were less than 11.

To identify the main factors associated with outpatient surgery, a mixed-effects, multilevel logistic regression model was developed for each operation where the outcome was inpatient vs outpatient surgery.^21^ Models included patient factors, hospital census division, and hospital characteristics. To account for clustering at the hospital level, hospital identifiers were included as a random intercept.^22,23^ To calculate attributable percentage of variability, 4 models were developed sequentially for each operation: null model 1 (with hospital as a random intercept); model 2, including model 1 plus patient characteristics; model 3, including model 2 plus hospital census division; and model 4, including model 3 plus the remainder of the hospital characteristics. Percentage of variance explained was derived from the covariance parameter estimate for the intercept compared with the covariance parameter estimate for the null model.^21^ In models where the covariance parameter estimate increased, covariates were removed 1 by 1 to determine the optimal model with the lowest covariance parameter estimate while retaining as many covariates as possible. Akaike information criterion values were also evaluated for each of the 4 models to ensure the model fit improved at each step of modeling. To account for multiple comparisons, the Benjamini and Hochberg method was applied to *P* values from the multilevel mixed-effects models to control for the false discovery rate.^24^

Two-sided *P* < .05 was considered statistically significant. Analyses were performed using SAS, version 9.4 (SAS Institute), and R, version 3.1.3 (R Foundation for Statistical Computing).

## Results

### Overall Cohort Description

A total of 456 954 patients were included, with 330 424 patients (72.3%) having undergone outpatient surgery. The median age of the cohort was 54 (IQR, 41-67) years. Among the 414 193 patients with data available, 268 692 (64.9%) were female and 145 501 (35.1%) were male. Total thyroidectomy was the most frequently performed outpatient surgery and MIS nephrectomy the least (eTable 2 in Supplement 1). From 2015 to 2021, 9 of 10 operations experienced increases in the rate of outpatient surgery (range, 2.6%-28.1%; *P* < .001). There was an 8.8% decline in the rate of outpatient open ventral hernia repair (*P* < .001). MIS ventral hernia repair increased by 7.3%, and 6 operations (simple mastectomy, mastectomy with implant-based reconstruction, MIS paraesophageal hernia repair, MIS nephrectomy, MIS hysterectomy, and MIS SO) experienced an increase of 10% or more during the study period.

For all 10 operations, the rate of outpatient surgery varied significantly by hospital census division (all *P* < .001). Hospitals in the Middle Atlantic region most frequently had the lowest rate of outpatient operations (open [60.9%] and MIS [73.1%] ventral hernia repair, MIS hysterectomy [26.3%], MIS SO [29.6%], and total thyroidectomy [76.5%]). Hospitals in the East South Central region most often had the highest rate of outpatient operations (simple mastectomy [89.1%], MIS cholecystectomy [82.0%], MIS ventral hernia repair [84.6%], MIS nephrectomy [29.0%], and total thyroidectomy [93.7%]). Mastectomy with reconstruction demonstrated the widest range for outpatient surgery (43.1% in New England vs 85.6% in Mountain region) (eFigure 1 in Supplement 1).

For most included operations, patients from metropolitan areas, those receiving care at hospitals with more beds, and those receiving care at hospitals with an approved residency training program were less likely to undergo outpatient surgery; exceptions were seen in MIS SO, simple mastectomy, and mastectomy with reconstruction (Table 1). Most patients (73.3%) had medical coverage for the full period of 365 days prior to the procedure; 96.9% of patients had medical coverage for the full 30 days following the procedure. Four of the 7 included operations demonstrated that hospitals with 400 or more beds had a lower likelihood of outpatient surgery (OR for MIS paraesophageal hernia repair, 0.58 [95% CI, 0.47-0.71; *P* < .001]; OR for MIS cholecystectomy, 0.73 [95% CI, 0.68-0.78; *P* < .001]; OR for open ventral hernia, 0.51 [95% CI, 0.46-0.57; *P* < .001]; OR for MIS ventral hernia repair, 0.66 [95% CI, 0.56-0.77; *P* < .001]).

**Table 1.** Distribution of Outpatient Surgery Rates by Patient and Hospital Factors

Factor	Rates of outpatient surgery, %									
	Simple mastectomy	Mastectomy with implant-based reconstruction	MIS paraesophageal hernia repair	MIS cholecystectomy	Open ventral hernia repair	MIS ventral hernia repair	MIS nephrectomy	MIS hysterectomy	MIS SO	Total thyroidectomy
Elixhauser comorbidities, No.										
0	93.8	83.5	75.4	85.1	90.8	92.8	19.0	46.5	55.0	94.9
1	82.3	77.1	70.3	81.4	82.3	89.2	23.9	41.8	46.3	92.2
2	83.3	74.5	68.5	79.2	74.3	86.2	22.8	39.5	40.9	89.0
3	82.8	72.9	65.5	76.3	64.6	81.8	20.9	35.7	32.7	87.7
4	81.1	74.1	62.0	73.3	56.3	77.5	19.5	30.9	26.9	84.6
5	80.7	72.3	60.3	70.2	49.5	72.2	19.3	33.5	17.1	84.4
6	78.6	73.1	59.8	67.1	43.6	68.7	17.9	30.5	13.3	83.6
7	79.5	68.8	58.2	62.8	40.1	64.4	16.3	36.1	25.6	79.0
8-14	77.0	67.7	52.8	55.8	31.9	57.8	14.7	27.8	<10.2	74.4
*P* value	<.001	<.001	<.001	<.001	<.001	<.001	<.001	<.001	<.001	<.001
RUCA										
Metropolitan	80.1	74.2	64.1	75.7	66.1	80.7	19.3	38.2	47.6	86.9
MSR	85.7	77.6	69.2	81.7	68.0	82.5	20.5	49.7	36.6	88.1
*P* value	<.001	<.001	<.001	<.001	<.001	.008	.18	<.001	<.001	.11
Bed size										
1-49	80.7	67.0	88.3	89.6	85.9	90.1	38.5	50.4	40.3	89.6
50-199	81.6	75.9	71.3	79.3	73.9	84.0	20.0	45.4	47.2	88.9
200-399	80.9	74.4	64.8	74.7	65.0	81.7	18.9	37.7	47.8	87.4
≥400	81.2	74.6	56.8	73.2	57.1	76.2	18.8	36.1	45.2	86.0
*P* value	.80	<.001	<.001	<.001	<.001	<.001	<.001	<.001	.03	<.001
Residency approved program										
Yes	81.6	75.3	60.9	75.2	62.8	79.4	19.2	38.1	47.1	86.4
No	80.1	71.2	74.1	79.0	72.9	83.9	20.5	43.3	44.4	89.0
*P* value	.02	<.001	<.001	<.001	<.001	<.001	.12	<.001	.03	<.001
Rural referral center										
Yes	84.1	75.1	61.3	77.1	61.0	82.7	21.5	46.9	44.6	86.8
No	80.7	74.4	65.5	76.7	67.0	80.8	19.2	39.6	46.5	87.1
*P* value	<.001	.50	<.001	.13	<.001	.01	.03	<.001	.35	.72
Outpatient rate change, 2015-2021^a^	12.7	27.7	18.8	2.6	−8.8	7.2	23.0	22.9	28.1	4.5
*P* value	<.001	<.001	<.001	<.001	<.001	<.001	<.001	<.001	<.001	<.001

Abbreviations: MIS, minimally invasive; MSR, micropolitan area core, small town core, or rural area; RUCA, rural-urban commuting area; SO, salpingo-oophorectomy.

^a^ Positive values denote increase in the rate of outpatient surgery from 2015 to 2021. Negative values indicate that the rate of outpatient surgery decreased.

### Adjusted Use of Outpatient Surgery 

On adjusted analysis, the likelihood of outpatient surgery varied Hospitals in the Middle Atlantic region were significantly less likely to perform outpatient operations compared with hospitals in New England (reference category), including MIS cholecystectomy (OR, 0.84; 95% CI, 0.72-0.97), open ventral hernia repair (OR, 0.80; 95% CI, 0.65-0.98), MIS hysterectomy (OR, 0.49; 95% CI, 0.32-0.76), and MIS SO (0.58; 95% CI, 0.36-0.95). In 7 of 10 operations, hospitals in the East South Central region were significantly more likely to be performed on an outpatient basis compared with hospitals in the New England region (OR range, 1.49 [95% CI, 1.06-2.10] for MIS ventral hernia repair to 5.67 [95% CI, 3.10-10.39] for mastectomy with reconstruction) (Table 2).

**Table 2.** Adjusted Odds of Outpatient Surgery by Hospital Census Division

Region	Simple mastectomy^a^		Mastectomy with implant-based reconstruction^b^		MIS paraesophageal hernia repair^c^		MIS cholecystectomy^d^		Open ventral hernia repair^d^		MIS ventral hernia repair^d^		MIS nephrectomy^e^		MIS hysterectomy^d^		MIS SO^f^		Total thyroidectomy^g^	
	OR (95% CI)	Adjusted *P* value^h^	OR (95% CI)	Adjusted *P* value^h^	OR (95% CI)	Adjusted *P* value^h^	OR (95% CI)	Adjusted *P* value^h^	OR (95% CI)	Adjusted *P* value^h^	OR (95% CI)	Adjusted *P* value^h^	OR (95% CI)	Adjusted *P* value^h^	OR (95% CI)	Adjusted *P* value^h^	OR (95% CI)	Adjusted *P* value^h^	OR (95% CI)	Adjusted *P* value^h^
MAT	0.69 (0.46-1.04)	.08	1.06 (0.63-1.78);	.82	1.11 (0.71-1.74)	.75	0.84 (0.72-0.97)	.03	0.80 (0.65-0.98)	.08	0.88 (0.65-1.20)	.49	1.29 (0.77-2.15)	.34	0.49 (0.32-0.76)	.006	0.58 (0.36-0.95)	.11	0.89 (0.54-1.47)	.65
ENC	3.52 (2.38-5.21)	<.001	6.23 (3.78-10.26)	<.001	1.67 (1.11-2.53)	.02	1.32 (1.14-1.51)	<.001	1.06 (0.88-1.28)	.54	1.29 (0.96-1.73)	.17	1.77 (1.09-2.87)	.03	0.99 (0.66-1.48)	.95	1.12 (0.71-1.77)	.92	2.03 (1.26-3.30)	.01
WNC	4.36 (2.85-6.67)	<.001	6.37 (3.69-10.26)	<.001	2.03 (1.31-3.15)	.003	1.54 (1.33-1.79)	<.001	0.94 (0.76-1.15)	.54	1.11 (0.77-1.44)	.74	1.91 (1.14-3.22)	.02	1.27 (0.83-1.96)	.44	1.03 (0.64-1.68)	.92	1.77 (1.06-2.96)	.06
SAT	2.96 (2.03-4.31)	<.001	7.29 (4.49-11.86)	<.001	2.16 (1.44-3.24)	<.001	1.12 (0.98-1.28)	.11	0.88 (0.73-1.06)	.34	1.35 (1.02-1.79)	.10	1.84 (1.15-2.94)	.02	0.83 (0.56-1.24)	.48	1.22 (0.78-1.91)	.76	1.93 (1.21-3.06)	.01
ESC	4.10 (2.58-6.53)	<.001	5.67 (3.10-10.39)	<.001	2.38 (1.47-3.84)	.001	1.68 (1.43-1.96)	<.001	0.93 (0.75-1.16)	.54	1.49 (1.06-2.10)	.10	3.55 (2.08-6.04)	<.001	0.85 (0.54-1.34)	.56	1.03 (0.61-1.72)	.92	3.74 (2.12-6.59)	<.001
WSC	2.23 (1.49-3.34)	<.001	5.09 (3.02-8.59)	<.001	2.83 (1.87-4.29)	<.001	1.18 (1.02-1.36)	.03	0.78 (0.64-0.94)	.04	1.37 (1.02-1.84)	.10	2.31 (1.41-3.77)	.003	0.71 (0.48-1.06)	.19	0.95 (0.60-1.50)	.92	1.41 (0.88-2.28)	.21
MTN	4.86 (3.04-7.76)	<.001	8.05 (4.64-13.98)	<.001	2.06 (1.31-3.26)	.003	1.16 (0.99-1.36)	.07	0.90 (0.73-1.12)	.54	1.15 (0.83-1.61)	.49	2.34 (1.37-4.00)	.005	1.49 (1.95-2.32)	.19	1.79 (1.10-2.90)	.11	1.15 (0.68-1.92)	.65
PCF	2.45 (1.58-3.79)	<.001	2.64 (1.55-4.49)	<.001	1.00 (0.63-1.58)	.98	0.57 (0.49-0.66)	<.001	0.72 (0.58-0.90)	.03	0.77 (0.55-1.09)	.23	1.67 (0.98-2.86)	.07	0.45 (0.29-0.70)	.003	0.64 (0.39-1.03)	.18	0.61 (0.36-1.02)	.09

Abbreviations: ENC, East North Central; ESC, East South Central; MAT, Middle Atlantic; MIS, minimally invasive; MTN, Mountain; PCF, Pacific; SAT, South Atlantic; WNC, West North Central; WSC, West South Central.

^a^ Model was adjusted for rural-urban commuting area (RUCA), bed size, residency training program, and rural referral center.

^b^ Model was adjusted for age, number of Elixhauser comorbidities, RUCA, bed size, and residency training program.

^c^ Model was adjusted for age, sex, number of Elixhauser comorbidities, RUCA, bed size, residency training program, and rural referral center.

^d^ Model was adjusted for procedure year, age, sex, number of Elixhauser comorbidities, RUCA, bed size, residency training program, and rural referral center.

^e^ Model was adjusted for age, sex, number of Elixhauser comorbidities, and RUCA.

^f^ Model was adjusted for age, number of Elixhauser comorbidities, and RUCA.

^g^ Model was adjusted for procedure year, age, sex, number of Elixhauser comorbidities, RUCA, and residency training program.

^h^ Adjusted *P* values use the Benjamini and Hochberg procedure to control the false discovery rate.

**Figure 2.**
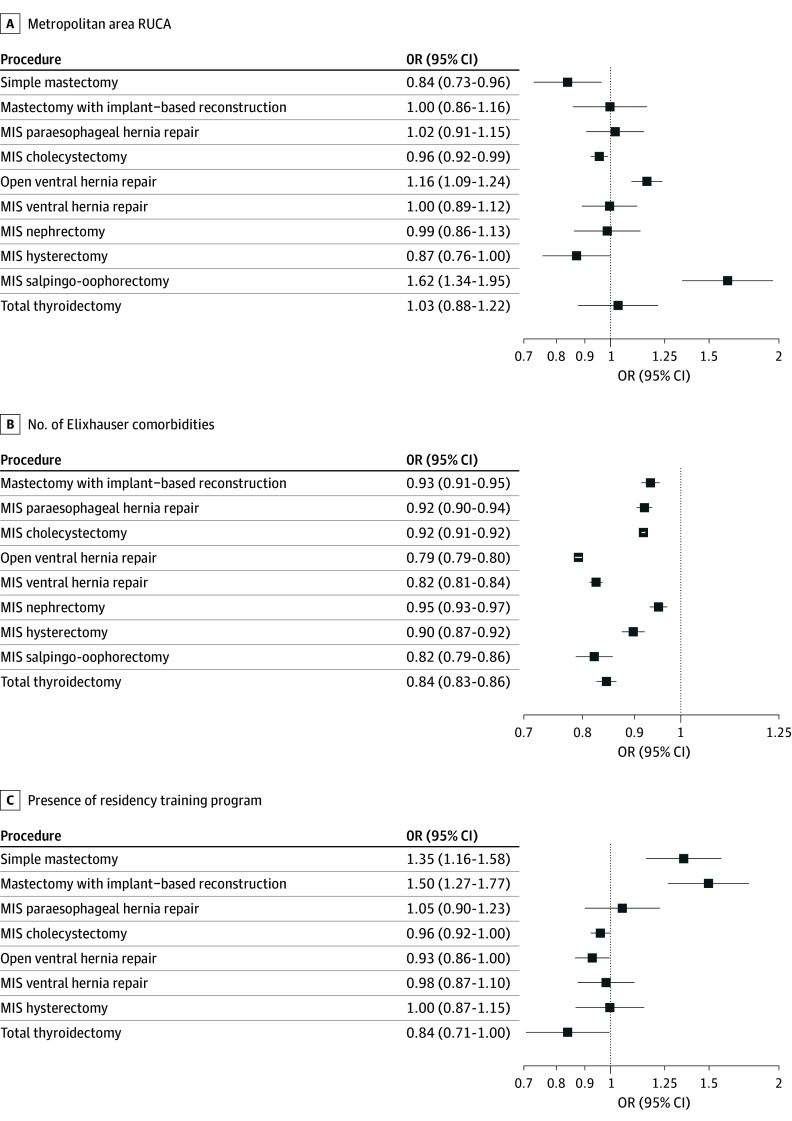
Odds of Outpatient Surgery Based on Patient and Hospital Characteristics Where the characteristic was not included in our final model for a given operation, the operation does not display for that characteristic. For metropolitan area, reference category was micropolitan area core, small town core, or rural area; for presence of residency training program, no program. Number of Elixhauser comorbidities was modeled as continuous, so odds ratio is calculated per increase of 1 comorbidity. MIS indicates minimally invasive; OR, odds ratio; and RUCA, rural-urban commuting area.

In terms of patient characteristics, the odds of undergoing outpatient surgery were significantly higher for patients from metropolitan areas for open ventral hernia repairs (OR, 1.16; 95% CI, 1.09-1.24) and MIS SO (OR, 1.62; 95% CI, 1.34-1.95) compared with being from small towns. The odds of outpatient simple mastectomy (OR, 0.84; 95% CI, 0.73-0.96) and MIS cholecystectomy (OR, 0.96; 95% CI, 0.92-0.99) were significantly lower in patients from metropolitan areas. They were no different for mastectomy with implant-based reconstruction (OR, 1.00; 95% CI, 0.86-1.16), MIS paraesophageal hernia repair (OR, 1.02; 95% CI, 0.91-1.15), MIS ventral hernia repair (OR, 1.00; 95% CI, 0.89-1.12), MIS nephrectomy (OR, 0.99; 95% CI, 0.86-1.13), and MIS hysterectomy (OR, 0.87; 95% CI, 0.76-1.00) (Figure 2).

Younger age was associated with lower odds of outpatient surgery for 8 of 9 operations (OR range, 0.95 [95% CI, 0.94-0.95] for MIS salpingo-oophorectomy to 0.99 [95% CI, 0.99-1.00] for mastectomy with reconstruction), and having fewer Elixhauser comorbidities was associated with lower odds of outpatient surgery for 9 of 9 operations (OR range, 0.93 [95% CI, 0.91-0.95] for mastectomy with implant-based reconstruction to 0.95 [95% CI, 0.93-0.97] for MIS nephrectomy) (Figure 2). Neither age nor Elixhauser comorbidities were included in the model for simple mastectomy.

The presence of an approved residency training program was associated with higher odds of outpatient simple mastectomy (OR, 1.35; 95% CI, 1.16-1.58; *P* < .001) and mastectomy with implant-based reconstruction (OR, 1.50; 95% CI, 1.27-1.77; *P* < .001) and lower odds of outpatient MIS cholecystectomy (OR, 0.96; 95% CI, 0.92-1.00; *P* = .04), open ventral hernia repair (OR, 0.93; 95% CI, 0.86-1.00; *P* = .04), and total thyroidectomy (OR, 0.84; 95% CI, 0.71-1.00; *P* = .04). Having a residency program did not change the rate for outpatient MIS paraesophageal hernia repair (OR, 1.05; 95% CI, 0.90-1.23), MIS hysterectomy (OR, 1.00; 95% CI, 0.87-1.15), or MIS ventral hernia repair (OR, 0.98; 95% CI, 0.87-1.10) (Figure 2).

### Attributable Variability

**Figure 3.**
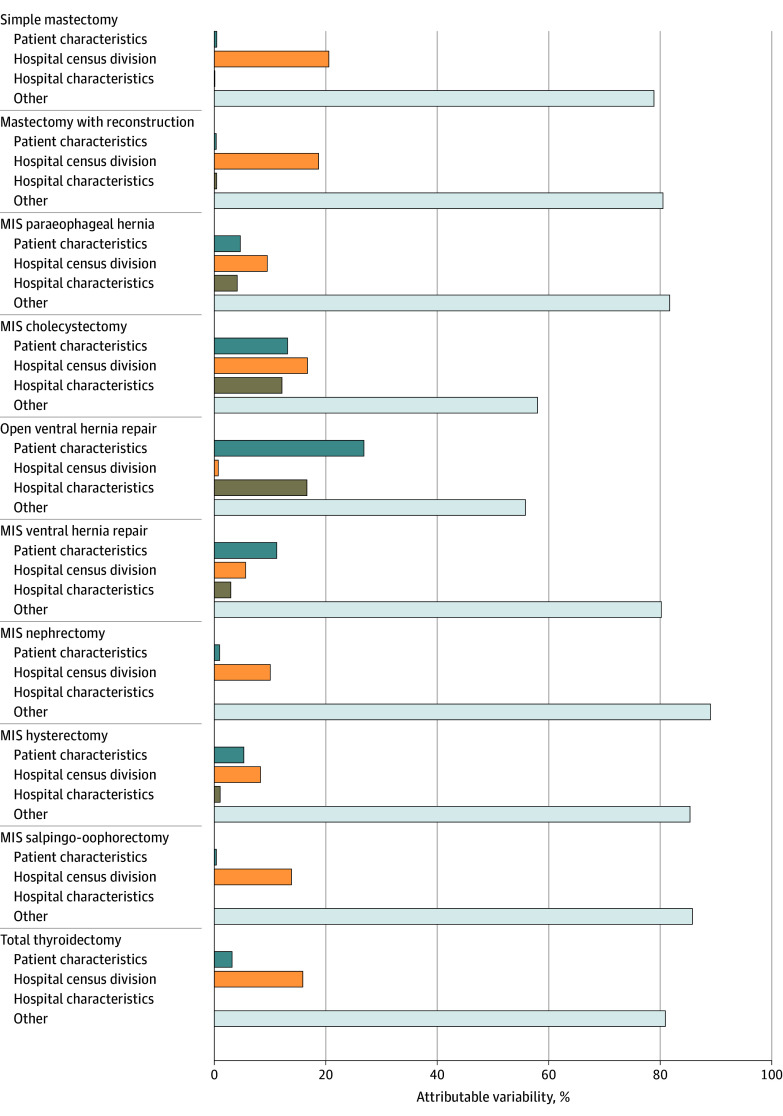
Percentage That Each Factor Contributed to the Variability in Outpatient Surgery MIS indicates minimally invasive.

Hospital census division explained the most variation in use of outpatient surgery in 8 of the 10 included operations (range, 8.3%-20.6%) (Figure 3). Hospital census division explained the greatest amount of outpatient surgery variation for mastectomy and mastectomy with reconstruction of all included procedures (20.6% and 18.7%, respectively). Patient characteristics explained the most variation in open ventral hernia repairs (26.9%) and MIS ventral hernia repairs (11.2%). Hospital characteristics explained the least amount of the variation (range, 0%-16.6%). In all 10 procedures, Akaike information criterion decreased from each sequential model to the next, indicating that the addition of patient characteristics, hospital census division, and the remainder of hospital characteristics improved the model fit compared with each previous model (eFigure 2 in Supplement 1).

## Discussion

Increased cost of delivering health care has been met with increasing demand to contain cost while maintaining quality of care.^25^ One way to reduce the cost of surgical care is through transitioning operations to the outpatient setting.^26,27^ Herein, we demonstrated an increase in the use of outpatient surgery from 2015 to 2021 for 9 of the 10 included operations across 456 954 patients with commercial insurance and Medicare Advantage coverage in the US. Notably, the use of outpatient surgery appeared to have significant unexplained geographic variation across the US, with the Northeast and Pacific Coastal regions having less frequent outpatient surgery that was not attributable to patient- or hospital-related factors. Other factors, including patients being from a metropolitan area, larger hospital size, and the hospital having a residency program, were to a lesser degree associated with lower rates of outpatient surgery, but the primary driver for most operations remained hospital geographic location (Figure 3). Improved understanding of practice variations and the sharing of practice protocols may engender quality improvement initiatives aimed at safety increasing outpatient surgery. These data should be used at the hospital and national levels to improve use of resources and reduce cost.

Interest in outpatient surgery was initially driven by improved patient satisfaction when patients were allowed to recover in a familiar environment as well as a general trend toward standardization of postsurgical care via Early Recovery After Surgery (ERAS) protocols.^28,29,30^ For example, ERAS protocols have not only demonstrated safety in same-day discharge but also are projected to save more than $700 000 per 1000 thyroidectomies.^31^ Pursuit of outpatient surgery is further substantiated by previous data from members of our group, which demonstrated lower rates of postoperative complications following outpatient surgery.^8^ While previous studies have demonstrated an overall increase in outpatient elective operations as well as their relative safety, the present study furthers the understanding of underlying currents that drive national practice variations.^8,27^ On multilevel analysis, hospital census division was the predominant contributor to outpatient surgery variation for 8 of 10 included operations. For example, 18.7% of the variation in outpatient mastectomy with implant-based reconstructions was explained by hospital census division, while only 0.8% of outpatient surgery variation was explained by patient and hospital factors. Additionally, when rates of outpatient operations were compared across individual hospital census locations, mastectomy with implant-based reconstructions experienced the greatest degree of variation, ranging from 43.1% being outpatient in the New England region to 85.6% being outpatient in the Mountain states. This geographically driven outpatient surgery practice pattern permeates across all 10 operations, as New England had the lowest rate for 4 operations and the Middle Atlantic states had the lowest outpatient surgery rate for 5 of the remaining 6 operations. This is in contrast to the East South Central and Mountain states, which had the highest rates of outpatient surgery.

While the multilevel analysis focused primarily on outpatient surgery practices based on hospital geography and not patient location, univariable analysis demonstrated similar results using patient address. This is consistent with previous studies demonstrating that most patients choose to undergo surgery based on geographic proximity (eFigure 3 in Supplement 1).^32^ Additional analysis showed that patient and hospital factors that are colloquially associated with urbanization, including being from a metropolitan area, increased hospital bed size, and the presence of surgical trainees, were all associated with lower rates of outpatient surgery.^33^ These 3 factors are also often associated with the performance of more complex operations that may not be amendable to outpatient surgery. To account for this, the analysis was only performed within each procedure and after applying strict exclusion criteria. As a result, the outpatient surgery association with geography is likely not attributable to variables outside of those included in the adjusted analysis, suggesting that the variation in outpatient surgery practices seen in this study cannot simply be explained by increased patient or case complexity.

We suspect that current outpatient surgery practice variations may be driven by surgeon dogma. Postoperative management, including the decision to perform an operation in the outpatient setting, is likely heavily influenced by the routine culture of practice that is specific to each hospital where the surgeon may work or have trained. Given the increased number of training programs in more densely populated coastal regions, and the rate at which surgeons practice in a geographic area close to where they trained, it is unlikely that we will see these geographic variations naturally align with time without external forces.^34,35^ Therefore, data such as those provided in this analysis are essential to help drive hospital and national practice trends, especially in the era of limited hospital resources and the increasing cost of care delivery. This is particularly important given the decreased rates of outpatient surgery seen at hospitals with residency programs, as these trainees will have less exposure to outpatient surgery.

The only exceptions to geography being the predominant contributor to outpatient surgery variation were open and MIS ventral hernia repair; for these procedures, patient characteristics outweighed hospital geography. Interestingly, while the rate of change of outpatient surgery by year was statistically significant across all included operations, when we compared the bookends of the study period (2015 vs 2021), open ventral hernia repair was the only procedure to show a decrease in outpatient surgery rates (8.7%), whereas outpatient surgery after MIS ventral hernia repair increased by a near commensurate amount (7.3%). These patterns likely represent outpatient surgery selection practices that are related to intrinsic patient characteristics. Due to their decreased postoperative pain and increased patient satisfaction benefits, an overall shift toward MIS techniques has occurred.^36^ Therefore, patients who remain in the open repair group likely represent those inappropriate for MIS. Further increases in outpatient surgery frequency may be difficult to achieve in ventral hernia repairs, and this may be why patient factors explained more of the existing practice variations than hospital census division for these procedures.

### Limitations

This study has limitations. Our findings are most applicable for patients with commercial insurance and Medicare Advantage coverage in the US. While we required medical coverage on the date of procedure for inclusion in the study, the lack of coverage within 365 days prior to the procedure could have contributed to an undercounting of Elixhauser comorbidities. However, given that 73.3% of the cohort had complete coverage within 365 days prior to procedure and Elixhauser comorbidities would likely have been captured in a presurgical assessment close to the date of surgery, gaps in coverage are unlikely to meaningfully change results. Additionally, we were unable to differentiate between scheduled and unscheduled cases, which could introduce some mixed-case complexity. Last, while hospital geography contributed the most to the variation in outpatient surgery practice of the patient and hospital factors that were identified, there may be other unknown factors that contribute to overall outpatient surgery variation. However, based on current geographical practice pattern variation, directed policy changes to standardize surgical practice nationally can be implemented.

## Conclusions

In this cross-sectional study, significant unexplained variation exists in use of outpatient surgery. Hospital geographic location was a more significant driver of the variation than patient or hospital characteristics. Using national benchmarks for use of outpatient surgery can help address practice variation, improve use of resources, and contain rising health care costs in the US.

## Notes

Supplement 1. **eTable 1.** *Current Procedural Terminology* codes and *International Classification of Diseases, Clinical Modification*, Codes Used to Identify Patients **eTable 2.** Distribution of Outpatient Surgery Over Time and by Procedure Type, Patient Demographics, and Hospital Geographic Region **eFigure 1**. Differences in the Rate of Outpatient Operations by Hospital Census Division **eFigure 2.** AIC Values During the Model Building Process for Attributable Variability Analysis **eFigure 3.** Differences in the Rate of Outpatient Operations by Patient Census Division

Supplement 2.
Data Sharing Statement
