# Supplementary material for: Variations in the Use of Outpatient Surgery
Source: JAMA Netw Open. 2025 Jul 31;8(7):e2524165. doi: 10.1001/jamanetworkopen.2025.24165 (PMC12314726; doi:10.1001/jamanetworkopen.2025.24165)
Supplement: Supplement 1. — eTable 1. Current Procedural Terminology codes and International Classification of Diseases, Clinical Modification, Codes Used to Identify Patients eTable 2. Distribution of Outpatient Surgery Over Time and by Procedure Type, Patient Demographics, and Hospital Geographic Region eFigure 1. Differences in the Rate of Outpatient Operations by Hospital Census Division eFigure 2. AIC Values During the Model Building Process for Attributable Variability Analysis eFigure 3. Differences in the Rate of Outpatient Operations by Patient Census Division [file jamanetwopen-e2524165-s001.pdf]

# Supplementary Online Content

Zhang C, Hanson K, Sangaralingham L, et al. Variations in the use of outpatient surgery. *JAMA Netw Open.* 2025;8(7):e2524165.
doi:10.1001/jamanetworkopen.2025.24165

**eTable 1.** *Current Procedural Terminology* codes and I*nternational Classification of Diseases, Clinical Modification,* Codes Used to Identify Patients
**eTable 2.** Distribution of Outpatient Surgery Over Time and by Procedure Type, Patient Demographics, and Hospital Geographic Region
**eFigure 1**. Differences in the Rate of Outpatient Operations by Hospital Census Division
**eFigure 2.** AIC Values During the Model Building Process for Attributable Variability Analysis
**eFigure 3.** Differences in the Rate of Outpatient Operations by Patient Census Division

This supplementary material has been provided by the authors to give readers additional information about their work.

© 2025 Zhang C et al. *JAMA Network Open*.

**eTable 1.** *Current Procedural Terminology* codes and *International Classification of Diseases, Clinical Modification,* Codes Used to Identify Patients

| Procedure | CPT codes | ICD-9-CM codes | ICD-10-CM codes |
|---|---|---|---|
| Simple mastectomy | 19303, 19304 | 85.34, 85.36, 85.41, 85.42 | 0HTT0ZZ, 0HTU0ZZ, 0HTV0ZZ |
| Mastectomy with implant-based reconstruction | At least one from both A and B:<br>(A) 19303, 19304, 19305, 19306, 19307<br>(B) 19340, 19342, 19350, 19351, 19355, 19357, 19360, 19361, 19364, 19366, 19367, 19368, 19369 | At least one from both A and B:<br>(A) 85.33, 85.34, 85.35, 85.36, 85.4x<br>(B) 85.33, 85.35, 85.51, 85.52, 85.53, 85.54, 85.70, 85.79, 85.86, 86.87, 85.89, 85.95 | At least one from both A and B:<br>(A) 07T50ZZ, 07T60ZZ, 07T80ZZ, 07T90ZZ, 0HTT0ZZ, 0HTU0ZZ, 0HTV0ZZ<br>(B) 0H0T0JZ, 0H0T0KZ, 0H0U0JZ, 0H0U0KZ, 0H0V0JZ, 0HHT0NZ, 0HHT3NZ, 0HHT7NZ, 0HHU0NZ, 0HHU3NZ, 0HHU7NZ, 0HHV0NZ, 0HHV3NZ, 0HHV7NZ, 0HHW0NZ, 0HHW3NZ, 0HHW7NZ, 0HHX0NZ, 0HHX3NZ, 0HHX7NZ, 0HNW0ZZ, 0HNX0ZZ, 0HNXXZZ, 0HQW7ZZ, 0HR5X74, 0HRT0JZ, 0HRU0JZ, 0HRU0KZ, 0HRV0JZ, 0HRW0KZ, 0HRWXJZ, 0HRX0JZ, 0HRXXJZ, 0HSXXZZ, 0HUW7JZ, 0HUW7KZ, 0HUW87Z, 0HUW8KZ, 0HUX0JZ, 0HUX0KZ, 0HUX7KZ, 0HUX8JZ, 0KXG0Z9 |
| MIS paraesophageal hernia repair | 43281, 43282, 43325, 43327 | 44.65, 44.66, 53.71 | 0DQ44ZZ, 0DU407Z, 0DU40JZ, 0DU40KZ, 0DU447Z, 0DU44JZ, 0DU44KZ, 0DV40CZ, 0DV40DZ, 0DV40ZZ, 0DV44ZZ |
| MIS cholecystectomy | 47562, 47563 | 51.23, 51.24, 87.53 | 0FT44ZZ, BF100ZZ, BF101ZZ, BF10YZZ |
| Open ventral hernia repair | 49560, 49565, 49570, 49585 | 53.41, 53.49, 53.51, 53.61, 53.69 | 0WQF0ZZ, 0WUF07Z, 0WUF0JZ, 0WUF0KZ |
| MIS ventral hernia repair | 49652, 49654, 49656 | 53.42, 53.43, 53.62, 53.63 | 0WUF4JZ |

© 2025 Zhang C et al. *JAMA Network Open*.

| | | | |
|---|---|---|---|
| MIS nephrectomy | 50543, 50545, 50546, 50549 | 40.3, 55.4, 55.51, | 07BC4ZZ, 0GT44ZZ, 0TB04ZZ, 0TB14ZZ, 0TB33ZZ, 0TB44ZZ, 0TL34CZ, 0TL44DZ, 0TT04ZZ, 0TT14ZZ |
| MIS hysterectomy | 58150, 58180, 58541, 58543, 58260, 58275, 58285, 58290 | 68.3x, 68.4x, 68.59, 68.79 | 0UT94ZZ, 0UT94ZZ, 0UT97ZL, 0UT97ZZ, 0UT98ZL, 0UT98ZZ, 0UT9FZL, 0UT9FZZ |
| MIS salpingo-oophorectomy | 58600, 58605, 58615, 58700, 58720, 58940, 59120, 59121, 59140, 59150, 59151 | 65.29, 65.39, 65.49, 65.51, 65.52, 65.61, 65.62, 66.01, 66.31, 66.32, 66.39, 66.4, 66.51, 66.52, 66.62, 66.63, 66.69, 66.92, 74.3 | 0U570ZZ, 0U573ZZ, 0U577ZZ, 0U9640Z, 0UB54ZZ, 0UB57ZZ, 0UB60ZZ, 0UB64ZZ, 0UB67ZZ, 0UB70ZZ, 0UB77ZZ, 0UC54ZZ, 0UC64ZZ, 0UC74ZZ, 0UL57DZ, 0UL60DZ, 0UL67DZ, 0UP837Z, 0UP83DZ, OUT0FZZ, 0UT54ZZ, 0UT5FZZ, 0UT64ZZ, 0UT6FZZ, 0UT74ZZ, 0UW840Z, 0UW84DZ, 10T24ZZ |
| Total thyroidectomy | 60240, 60252 | 6.4 | 0GTK0ZZ |

ICD-9-CM, International Classification of Diseases, Clinical Modification, Ninth Revision; ICD-10-CM, International Classification of Diseases, Clinical Modification, Tenth Revision, CPT, Current Procedural

© 2025 Zhang C et al. *JAMA Network Open*.

**eTable 2.** Distribution of Outpatient Surgery Over Time and by Procedure Type, Patient Demographics, and Hospital Geographic Region

| | Outpatient vs inpatient | | |
|---|---|---|---|
| | Inpatient (N=126,530) | Outpatient (N=330,424) | p-value |
| **Procedure year**, n (%) | | | <0.001 |
| 2015 | 22,194 (32.3%) | 46,551 (67.7%) | |
| 2016 | 20,405 (28.0%) | 52,389 (72.0%) | |
| 2017 | 20,932 (28.2%) | 53,368 (71.8%) | |
| 2018 | 20,260 (27.5%) | 53,381 (72.5%) | |
| 2019 | 19,355 (26.4%) | 54,056 (73.6%) | |
| 2020 | 15,355 (25.3%) | 45,456 (74.7%) | |
| 2021 | 8,029 (24.1%) | 25,223 (75.9%) | |
| **Operation**, n (%) | | | |
| Simple mastectomy | 3,606 (18.8%) | 15,530 (81.2%) | <0.001 |
| Mastectomy with recon | 5,710 (25.5%) | 16,659 (74.5%) | |
| MIS paraesophageal hernia | 5,264 (35.0%) | 9,790 (65.0%) | |
| MIS cholecystectomy | 60,927 (23.2%) | 201,506 (76.8%) | |
| Open ventral hernia repair | 21,183 (33.6%) | 41,929 (66.4%) | |
| MIS ventral hernia repair | 4,480 (19.0%) | 19,119 (81.0%) | |
| MIS nephrectomy | 11,499 (80.6%) | 2,776 (19.4%) | |
| MIS hysterectomy | 7,452 (59.8%) | 5,006 (40.2%) | |
| MIS salpingo-oophorectomy | 4,259 (53.7%) | 3,675 (46.3%) | |
| Total thyroidectomy | 2,150 (13.0%) | 14,434 (87.0%) | |
| **Age**, median (IQR) | 60 (46, 71) | 52 (39, 65) | <0.001 |
| **Sex**, n (%) | | | |
| Male | 44,324 (30.5%) | 101,177 (69.5%) | <0.001 |
| Female | 64,785 (24.1%) | 203,907 (75.9%) | |
| Missing | 17,421 | 25,340 | |
| **Census division (hospital), n (%)** | | | |
| New England | 5,562 (33.0%) | 11,307 (67.0%) | <0.001 |
| Mid Atlantic | 13,621 (38.6%) | 21,703 (61.4%) | |
| East North Central | 18,405 (25.1%) | 54,937 (74.9%) | |
| West North Central | 12,214 (24.3%) | 38,053 (75.7%) | |
| South Atlantic | 33,864 (28.6%) | 84,450 (71.4%) | |
| East South Central | 6,616 (22.7%) | 22,575 (77.3%) | |
| West South Central | 19,486 (26.6%) | 53,653 (73.4%) | |
| Mountain | 8,618 (22.9%) | 29,093 (77.1%) | |
| Pacific | 8,144 (35.7%) | 14,653 (64.3%) | |

*Mastectomy with recon: Mastectomy with implant-based reconstruction; MIS: minimally invasive surgery*
*P-values are from chi-square tests for discrete variables and Kruskal-Wallis tests for continuous variables.*

© 2025 Zhang C et al. *JAMA Network Open*.

**eFigure 1**. Differences in the Rate of Outpatient Operations by Hospital Census Division

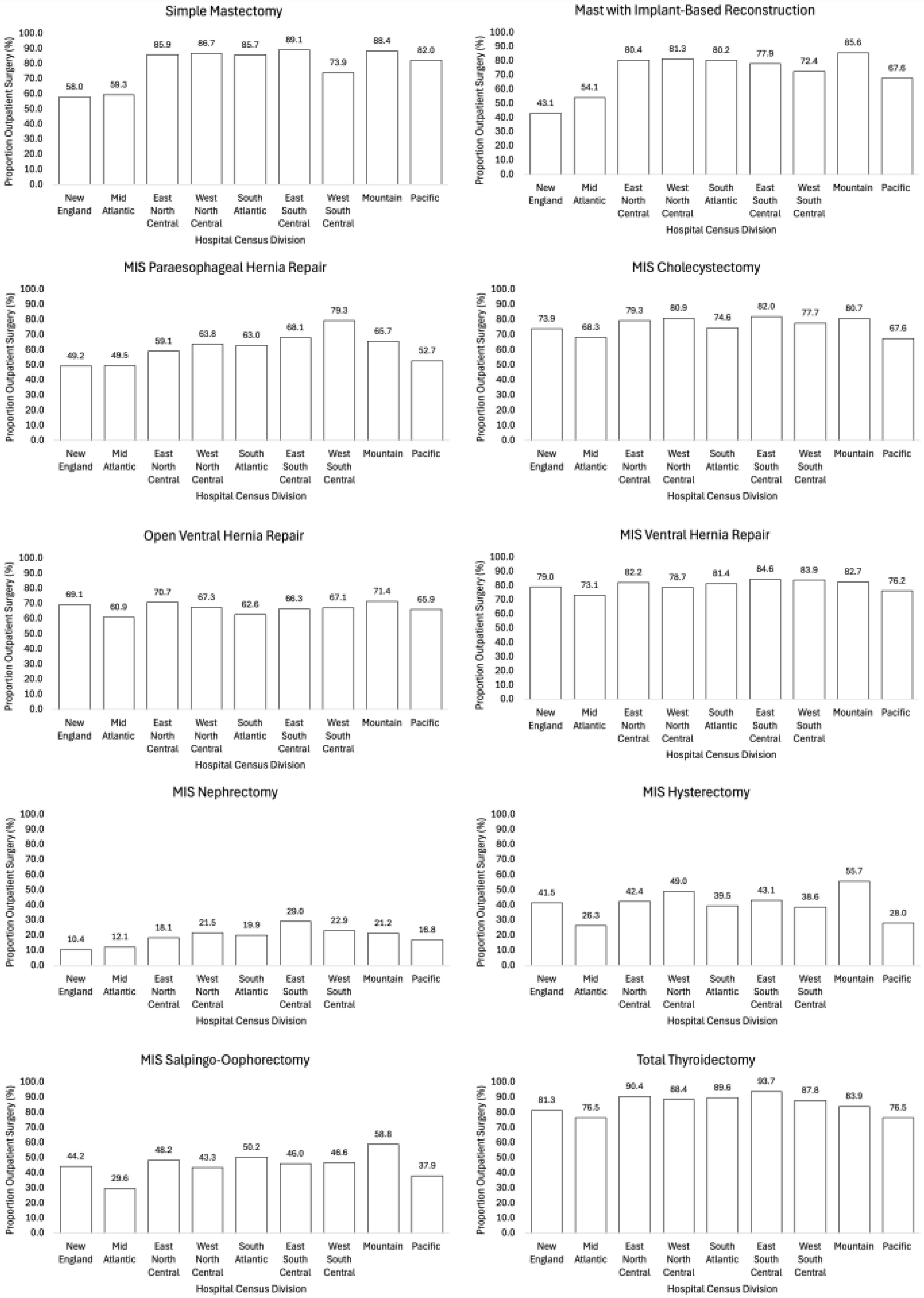


© 2025 Zhang C et al. *JAMA Network Open*.

**eFigure 2.** AIC Values During the Model Building Process for Attributable Variability Analysis

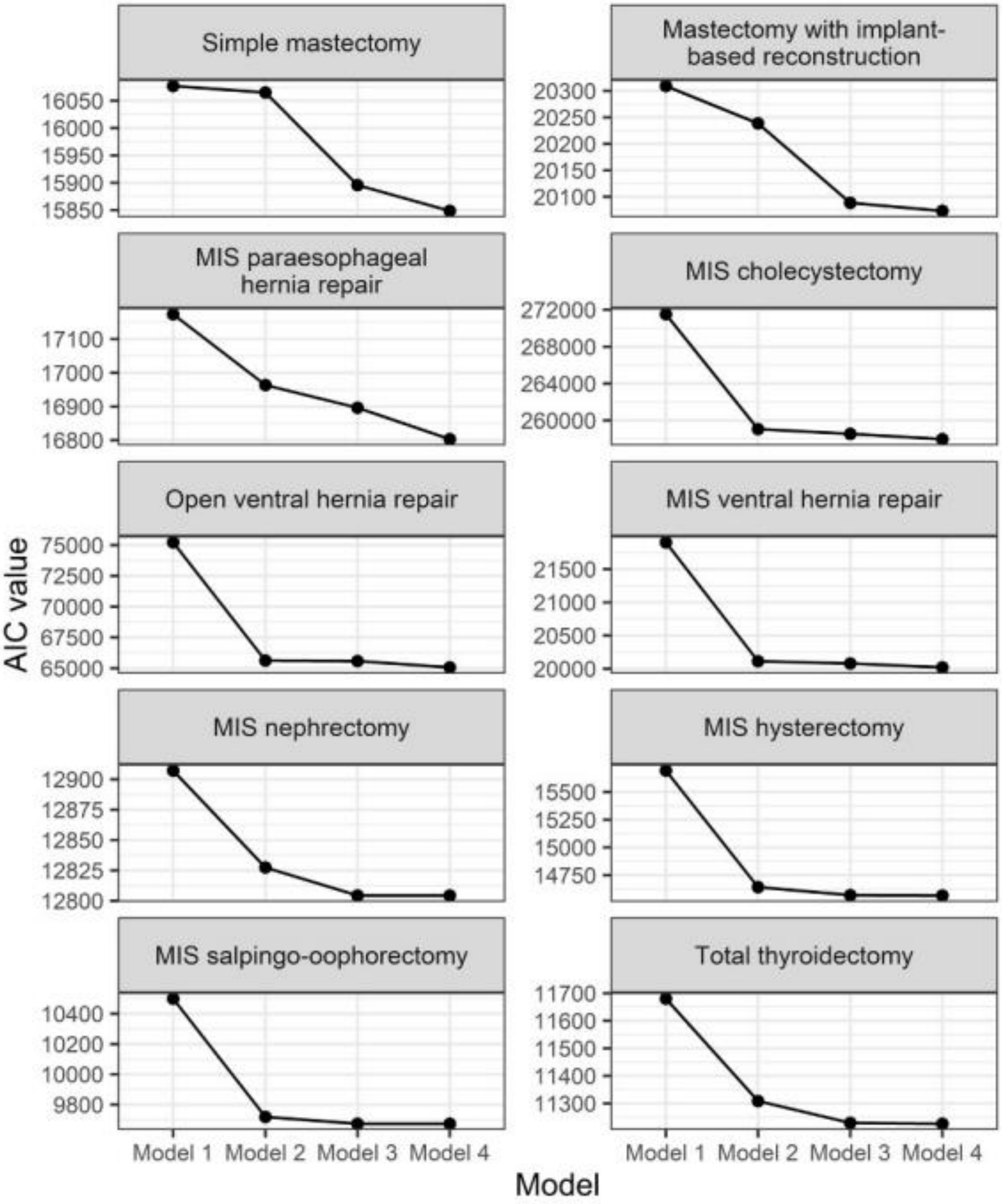


Smaller AIC indicates better model fit. Model 1: null model with hospital as a random intercept; Model 2: model 1 plus patient characteristics; Model 3: model 2 plus hospital census division; Model 4: model 3 plus the remainder of the hospital characteristics.

© 2025 Zhang C et al. *JAMA Network Open*.

**eFigure 3.** Differences in the Rate of Outpatient Operations by Patient Census Division

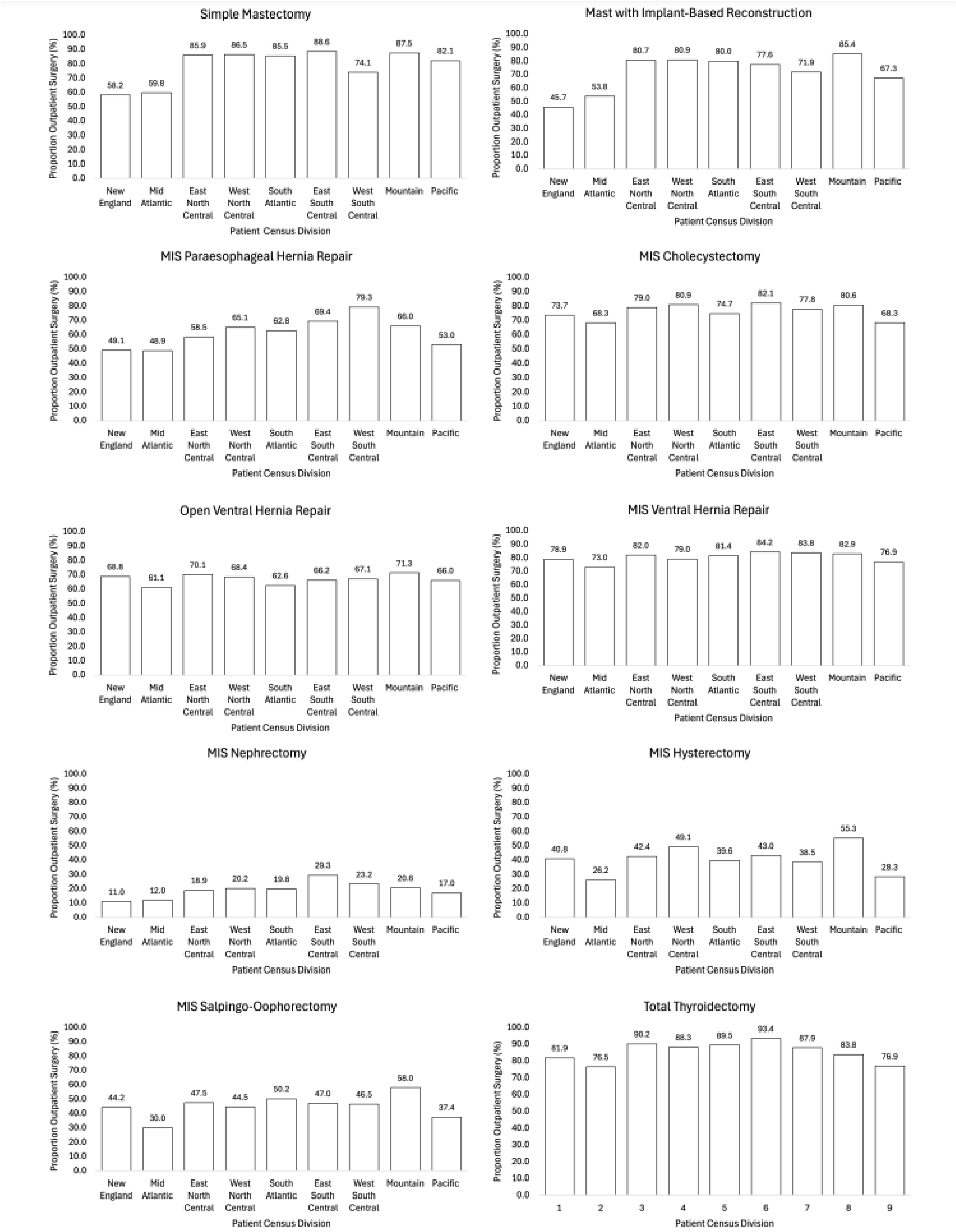


© 2025 Zhang C et al. *JAMA Network Open*.
