# Supplementary material for: Variations in the Use of Outpatient Surgery
Source: JAMA Netw Open. 2025 Jul 31;8(7):e2524165. doi: 10.1001/jamanetworkopen.2025.24165 (PMC12314726; doi:10.1001/jamanetworkopen.2025.24165)
Supplement: Supplement 2. — Data Sharing Statement [file jamanetwopen-e2524165-s002.pdf]

Data Sharing Statement

Zhang. Variations in the Use of Outpatient Surgery. *JAMA Netw Open*. Published July 31, 2025. doi:10.1001/jamanetworkopen.2025.24165

Data
Data available: No

Additional Information
Explanation for why data not available: Optum Datawarehouse is a national database with de-identified patient data.
